# Research of a Novel 3D Printed Strain Gauge Type Force Sensor †

**DOI:** 10.3390/mi10010020

**Published:** 2018-12-29

**Authors:** Mingjie Liu, Qi Zhang, Yiwei Shao, Chuanqi Liu, Yulong Zhao

**Affiliations:** State Key Laboratory for Manufacturing Systems Engineering, Xi’an Jiaotong University, Xi’an 710049, China; liumingjie@stu.xjtu.edu.cn (M.L.); imporeed@stu.xjtu.edu.cn (Y.S.); liuchuanqi@stu.xjtu.edu.cn (C.L.)

**Keywords:** 3D printed force sensor, digital light processing, inkjet printing, sensor fabrication

## Abstract

A 3D printed force sensor with a composite structure developed by combining digital light processing (DLP) based printing and inkjet printing technologies is described in this paper. The sensor has cost effectiveness and time-saving advantages compared to the traditional sensor manufacturing process. During this work, the substrate of the force sensor was printed by a DLP based 3D printer using a transparent high-temperature resin, and the strain gauge of the force sensor was inkjet printed using poly(3,4-ethylenedioxythiophene) polystyrene sulfonate (PEDOT/PSS) conductive ink. Finite element (FE) simulation was conducted to find the print origin of the strain gauge. The relationship between the mechanical properties of the post-cured resin and the curing time was investigated and the resistance of the printed strain gauges was characterized to optimize process parameters. Afterward, the force sensor was characterized. Experimental results show that the sensitivity of the sensor is 2.92% N^−1^ and the linearity error is 3.1485% full scale (FS) within the range from 0 mN–160 mN, and the effective gauge factor of the strain gauge is about 0.98. The resistance drifting is less than 0.004 kΩ within an hour. These figures prove that the device can perform as a force sensor and 3D printing technology may have great applied potential in sensor fabrication.

## 1. Introduction

The device that can convert the applied force into related electrical signals is called a force sensor. It usually works by measuring the displacement or strain of the force sensing element, which is called a flexure [1]. Force sensors are widely used in a large range of devices and systems, such as transportation, robotics, manufacturing industry, and healthcare technologies. Typical force sensors are usually fabricated with micro-electromechanical systems (MEMS) technology, which has the disadvantages of limited range of functional materials, in addition to being fragile and expensive. There is a growing need for inexpensive and easily customized force sensors where the force information is very important but high-precision measurements are not required [1].

3D printing, also known as additive manufacturing, is an emerging technology for sensor fabrication with economical, timesaving, geometrically complex and functionally complex advantages. It works by printing multiple layers of material under computer control to create three-dimensional physical objects. In recent years, a considerable amount of research has appeared in the fields of mechanics, electronics, kinematics, optics, acoustics, etc. 3D printing is particularly suitable for the fabrication of force sensing modules and electronic modules of the sensors [2,3]. However, in view of this research on 3D printed force sensors, most of it only uses 3D printing technology to fabricate force sensing elements and packages of the sensors, and it is often necessary to use commercial strain gauges [4,5,6] and optical fibers [1,7,8] to realize signal conversion and measurement. The bonding of the strain gauge and the installation of the optical fiber also brings alignment errors and restricted sensors performance [1,9]. Moreover, most of this research only uses one kind of 3D printing technology and a single raw material [10,11,12], and these methods are difficult to use to form a composite structure. Although Eijking and Schouten et al. [13,14] have used fused deposition manufacturing (FDM) technology to fabricate a series of force sensors with different materials to form a composite structure, the precision of FDM technology is low [2] and the fabricated sensor structure is rough, making it difficult to fabricate high-precision micro-nano sensors. The use of a single 3D printing technology also limits the application of 3D printed materials.

In view of the above points, a completely 3D printed force sensor with a composite structure based on the combination of digital light processing (DLP) based printing and inkjet printing technologies is proposed in this paper. The DLP based printing can produce complex structures with high precision, and the inkjet printing has advantages featuring maskless, non-contact, and simple process steps compared with screen printing and spin coating [15,16,17]. Theoretical and finite element method (FEM) models were established to analyze the sensor structure. The substrate of the force sensor was made from a transparent high-temperature resin, and the strain gauge was directly inkjet printed on the sensor substrate using poly(3,4-ethylenedioxythiophene) polystyrene sulfonate (PEDOT/PSS) conductive ink. It only took a few hours to fabricate the force sensor with the use of 3D printing technologies. The method mentioned in this paper avoids the bonding of the strain gauge and has timesaving and economical advantages. Finally, experiments were done to characterize the strain gauge and the force sensor.

## 2. Design and Modeling 

### 2.1. Sensor Design

**Figure 1:**
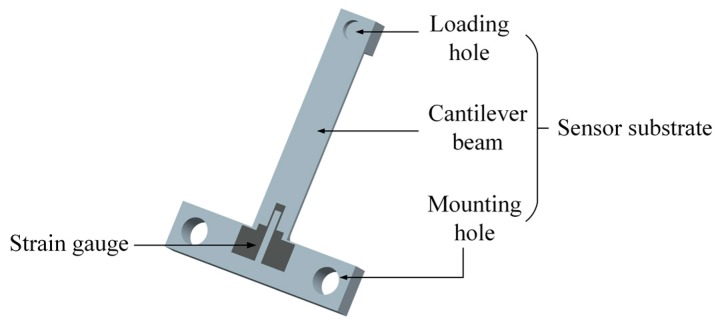
Diagrammatic sketch of the proposed force sensor.

**Figure 2:**
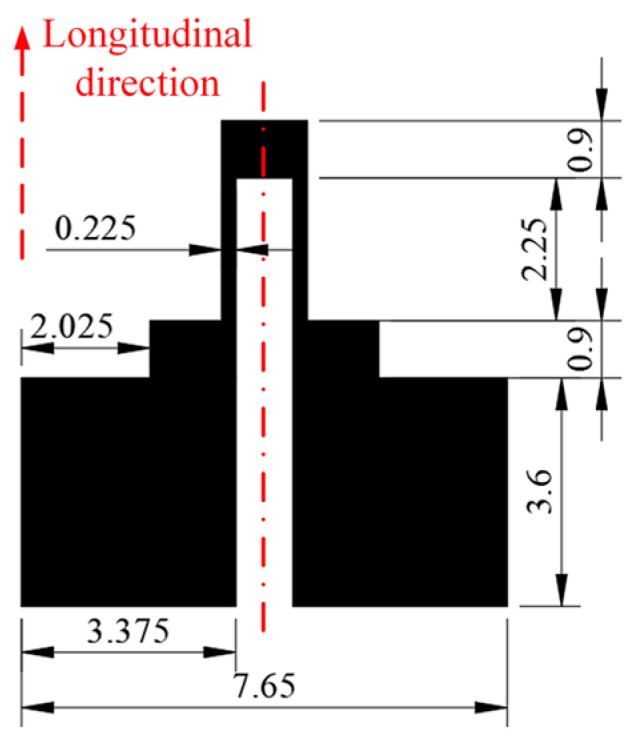
The dimensions of the strain gauge. Units: mm.

The proposed force sensor is schematically shown in Figure 1. The force sensor is composed of a T-shape substrate and a strain gauge, which located at one end of the substrate. There is a loading hole and two mounting holes on the substrate and the main body of the substrate is a cantilever beam, which is designed as the flexure of the force sensor. There is a rounded corner designed at the end of the cantilever beam near the mounting hole to protect the substrate from breakage during bearing the load. The cantilever beam is 25 mm long, 5 mm wide, and 0.5 mm thick. The radius of the rounded corner is 1 mm and the dimensions of the strain gauge are shown in Figure 2. The sensor structure is simple. This is because the purpose of this paper is to show the feasibility of combining DLP based printing and inkjet printing technologies to fabricate force sensors. The sensor substrate is designed as a T-shape for clamping easily during the experiments, and the mounting holes are reserved for future packaging.

**Figure 3:**
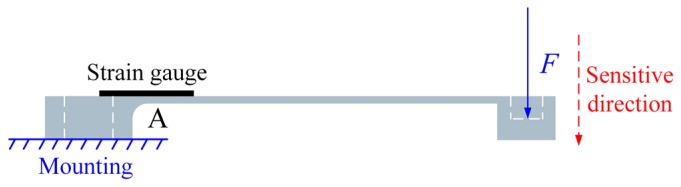
The working principle of the force sensor.

As shown in Figure 3, when the force is applied at the loading hole, the cantilever beam deforms and a maximal strain occurs near the fixed end of the cantilever beam, i.e., position A. The function of the strain gauge printed at position A converts the strain change to a resistance variation. The strain gauge is thin and the adherence between the strain gauge and the cantilever beam is perfect. In those conditions, the strain of the strain gauge is approximately equal to the strain of the cantilever beam [18].

### 2.2. Theoretical Model

As shown in Figure 3, the maximum longitudinal strain induced by the applied force can be written:

(1) $$\varepsilon_{l\max}=\frac{Flt}{2EI}$$

where

(2) $$I=\frac{wt^{3}}{12}$$

where $\varepsilon_{l\max}$ is the maximum strain; *E* is the Young’s modulus of the cantilever beam; *F* is the applied force; *I* is the moment of inertia with respect to the neutral axis; and *l*, *w*, and *t* are the length, width and thickness of the cantilever beam, respectively. Substituting Equation (2) into Equation (1), the maximum strain can be expressed as:

(3) $$\varepsilon_{l\max}=\frac{6Fl}{Ewt^{2}}$$

The maximum stress for the cantilever beam also occurs at the fixed end, which can be expressed as:

(4) $$\sigma_{l\max}=E\varepsilon_{l\max}=\frac{6Fl}{wt^{2}}$$

where $\sigma_{l\max}$ is the maximum stress.

The resistance variation of the strain gauge induced by the applied force can be written as [19]:

(5) $$\frac{\Delta R}{R}\;=\;G_{e}\varepsilon_{l\max}\;=\;G_{e}\frac{6Fl}{Ewt^{2}}$$

Here, *R* is the initial resistance value of the strain gauge; Δ*R* is the change of *R*; *G_e_* is the effective gauge factor of the strain gauge. Equation (5) indicates that the relative resistance change of the strange gauge is linearly related to the applied force. So by measuring the resistance change of the strain gauge, the magnitude of the applied force can be calculated.

**Figure 4:**
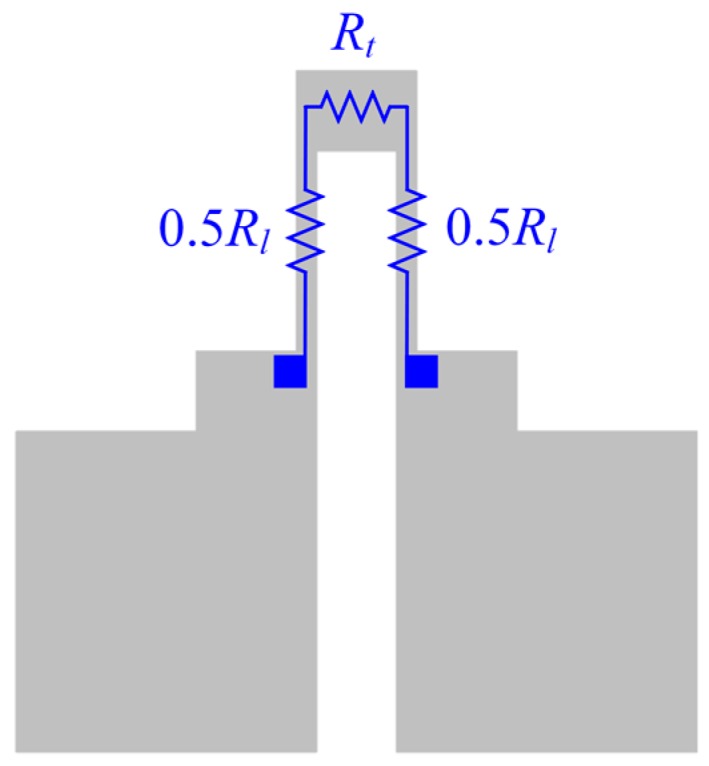
A schematic diagram of the resistance of the strain gauge.

The resistance of the strain gauge can be divided into the longitudinal branch resistance *R_l_* and the transversal branch resistance *R_t_*, as shown in Figure 4. The relationship between the effective gauge factor of the strain gauge and the intrinsic gauge factor of the material can be written as [18]:

(6) $$G_{e}=G_{m}\frac{1-\nu \alpha }{1+\alpha }$$

where *G_m_* is the intrinsic gauge factor of the material; *ν* is the Poisson ratio and *α* is the ratio between *R_t_* and *R_l_*.

According to the dimensions of the strain gauge, the ratio between *R_t_* and *R_l_* can be calculated, resulting in *α* = 0.075. Considering a Poisson ratio *ν* = 0.35, Equation (6) can be approximated as:

(7) $$G_{e}=0.91G_{m}$$

Equation (7) shows that the effective gauge factor of the strain gauge is close to the intrinsic gauge factor of the material, so the design of the strain gauge in this paper is reasonable.

### 2.3. Simulation Analysis

**Figure 5:**
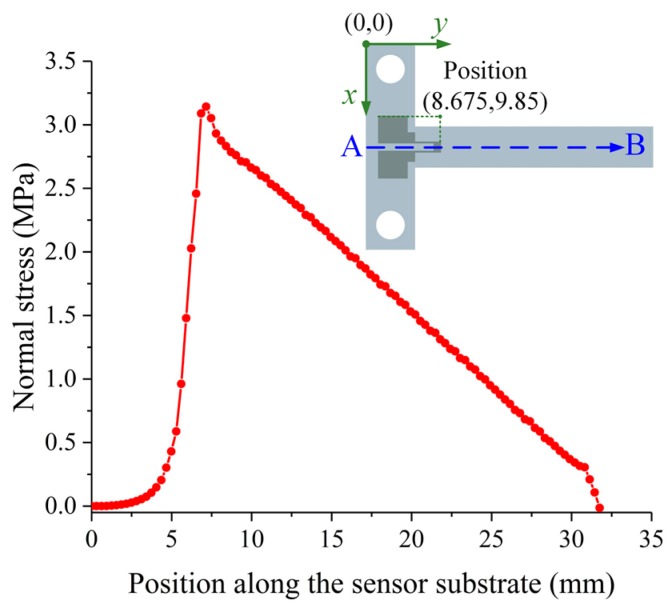
The normal stress distribution along the path A to B on the sensor substrate and the position of the strain gauge.

The position of the strain gauge was determined using the finite element method (FEM). The normal stress distribution along the sensor substrate was investigated under a loading force *F*. The sensor substrate has an elastic modulus (*E*) of 3.6 GPa and a Poisson ratio (*ν*) of 0.35. The cantilever beam was protected by a 60 µm thick Kapton tape pasted on it, which has an elastic modulus (*E*) of 3 GPa and a Poisson ratio (*ν*) of 0.35. The sensor substrate with the Kapton tape was analyzed using ANSYS Workbench 14.5 from ANSYS (Ansys Inc., Canonsburg, PA, USA). The contact type between the Kapton tape and the sensor substrate was set to be bonded. The mesh was generated automatically. A 50 mN loading force was applied at the load hole and the mounting holes were fixed and supported. It can be seen from the results that the maximum normal stress for the cantilever beam with a value of 3.1427 MPa occurs at the fixed end, and the normal stress distribution along the sensor substrate from point A to point B is shown in Figure 5. Due to the fact that the longitudinal branches of the strain gauge should be located where the normal strain is big and the normal strain is proportional to the normal stress, the position of the strain gauge was determined at the position (8.675, 9.85), as shown in Figure 5.

## 3. Fabrication of the Force Sensor

The processing of the force sensor mainly consists of two major steps, the fabrication of the sensor substrate and the fabrication of the strain gauge. A PEDOT/PSS conductive ink was used to fabricate the strain gauge. This is because PEDOT/PSS conductive ink has advantages such as high conductivity, good stability [20], and easy to clean and hard to clog nozzles, and it has piezoresistive properties [16,17]. The PEDOT/PSS conductive ink needs to be dried in an air convection oven at 80 °C–130 °C for 15 min to make the PEDOT/PSS functional. However, the ordinary photosensitive resin cannot meet the needs of this process due to the low heat deflection temperature. Therefore, a transparent high-temperature resin with a 289 °C heat deflection temperature from Formlabs (Somerville, MA, USA) was used to fabricate the sensor substrate.

### 3.1. Fabrication and Characterization of the Sensor Substrate

**Figure 6:**
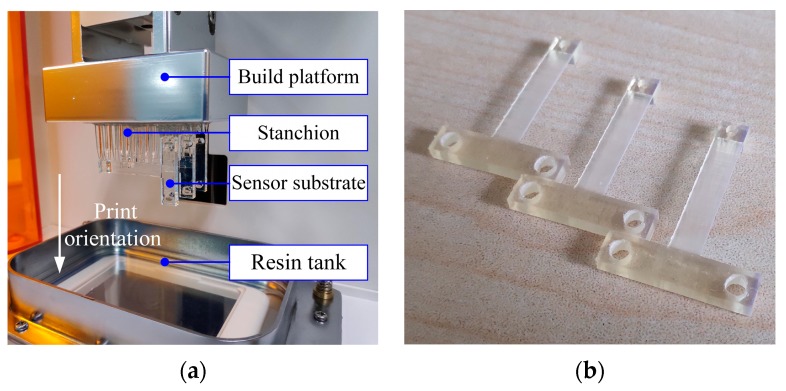
Pictures of the sensor substrates: (**a**) After printing; (**b**) after ultraviolet curing.

The substrate of the force sensor was printed by M-Jewelry U50 printer from MAKEX (Makex CO., LTD., Ningbo, China) [21]. It is a DLP based desktop 3D printer with a lateral resolution of 50 µm and a thickness resolution of 5 µm. During printing, the layer thickness to form the structure was set to 30 µm. The sensor substrates after printing are shown in Figure 6a.

**Figure 7:**
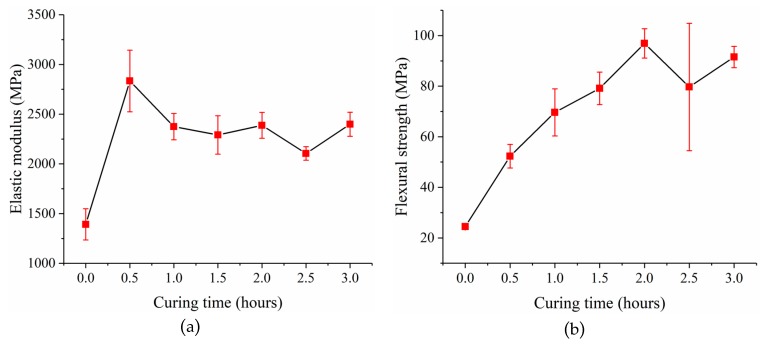
(**a**) The elastic modulus of the post-cured resin versus curing time; (**b**) the flexural strength of the post-cured resin versus curing time.

After that, the printed sensor substrate was ultraviolet (UV) cured in a UV curing machine from MAKEX [22] to improve the mechanical properties of the post-cured resin. Experiments were done to investigate the relationship between the mechanical properties of the post-cured resin and the curing time. First, thirty-five resin samples that were 60 mm long, 15mm wide and 3 mm thick were prepared and divided into 7 groups. Each group was UV cured with different curing times ranging from 0 h to 3 h. The light intensity in the UV curing machine is (25.34 ± 1.39) × 103 Lux, which was measured by an illuminometer GM1040 from BENETECH (Shenzhen, China). Finally, a three-point bending test was carried out with these samples using a multifunctional static test machine CMT4304 from MTS (Eden Prairie, MN, USA). The elastic moduli and flexural strengths of these samples were obtained. The average value and standard deviation of the experimental data are listed in Table 1. The relationships between the elastic modulus of the post-cured resin and the curing time, and the flexural strength of the post-cured resin and the curing time are shown in Figure 7a,b, respectively. The results show that the post-cured resin with 2 h curing time has relatively better mechanical properties with an elastic modulus of 2388.12 ± 225.76 MPa and flexural strength of 96.91 ± 10.05 MPa. The flexural strength of the samples experienced a large standard deviation for curing time at 2.5 h. This is because of the resin aging due to the long curing time, which makes its performance unstable [23]. So, the sensor substrate was cured for 2 h to get better mechanical properties. The sensor substrates after ultraviolet curing are shown in Figure 6b.

Due to the large surface roughness of the sensor substrate, a polishing step was needed to make the substrate meet the requirements of the follow-up process. The sensor substrate was polished with 2000 mesh, 3000 mesh, 5000 mesh and 7000 mesh sandpapers in sequence. Finally, the roughness of the substrate was reduced from 0.4541 ± 0.1640 µm to 0.0516 ± 0.0072 µm and it was smooth enough to meet the needs of the inkjet printing process. The sensor substrate was then treated with the oxygen plasma in a plasma cleaner for 1 min to make it clean and hydrophilic.

### 3.2. Fabrication and Characterization of the Strain Gauge

The strain gauge was printed by a commercialized drop-on-demand (DOD) Dimatix DMP-3000 material inkjet printing system from Fujifilm (Tokyo, Japan) with a DMC-11610 cartridge consisting of 16 nozzles and a drop volume of 10 pL. A commercialized PEDOT/PSS ink CLEVIOS P Jet 700 N from Heraeus (Hanau, Germany) [24] with 0.6–1.2 wt% of solids content and 659 S/cm conductivity was used to make the strain gauge. The PEDOT/PSS ink was printed with a 45 µm drop space using a frequency of 5 kHz at a voltage of 38 V and a meniscus vacuum set to 4 inches of H_2_O. During printing, the droplets were ejected out of the nozzle by applying a voltage that changed the volume of the channel connecting to the nozzle. The voltage waveforms used during printing are listed in Table 2. In addition, other printing details followed the inkjet guide, *Inkjet printing with Clevios P Jet formulations*, provided by Heraeus. Next, the samples were dried in an air convection oven at 120 °C for 15 min to make the strain gauge functional. 

**Figure 8:**
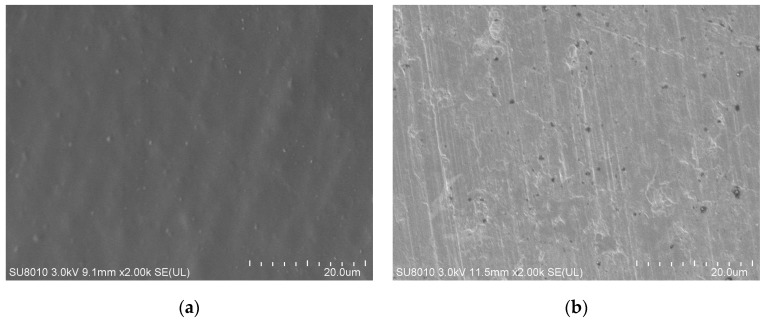
Scanning electron microscope images of the PEDOT/PSS film: (**a**) Dried at the room temperature for 24 h; (**b**) dried in an air convection oven at 120 °C for 15 min.

**Figure 9:**
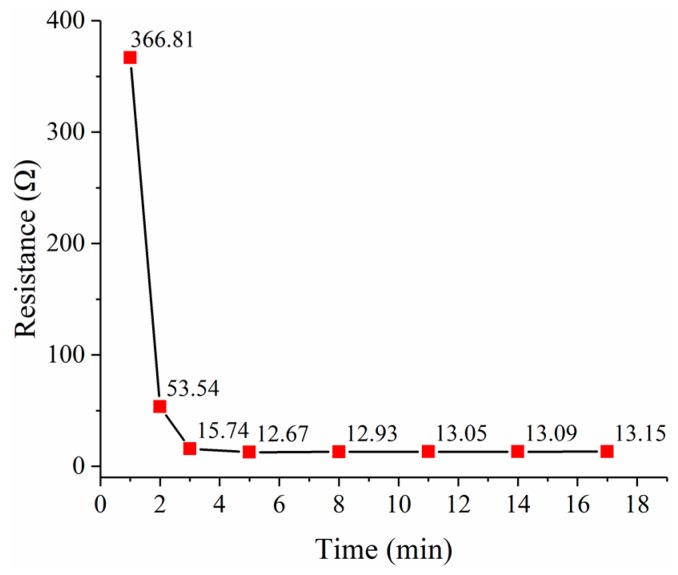
The resistance of the PEDOT/PSS film versus the heat drying time.

The PEDOT/PSS films dried at the room temperature for 24 h and dried in an air convection oven at 120 °C for 15 min were observed using a scanning electron microscope SU-8010 from HITACHI (Tokyo, Japan). It can be seen that the heat drying process can make the PEDOT/PSS film flatter and denser, as shown in Figure 8. The effect of the heat drying process on the PEDOT/PSS film resistance was also investigated, as shown in Figure 9. The resistance of the PEDOT/PSS film decreased rapidly when heat drying begins and tended to be stable after 8 min.

**Figure 10:**
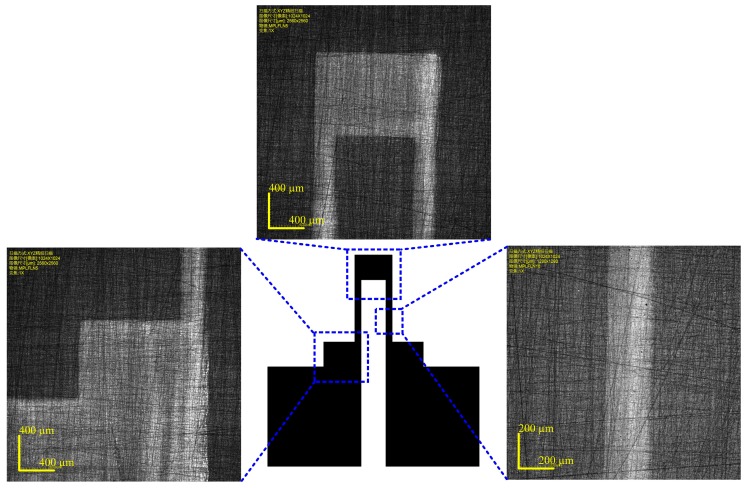
Confocal laser scanning microscope images of the strain gauge.

In order to study the morphology of the printed strain gauge, the printed strain gauge with three layers of PEDOT/PSS on the sensor substrate was observed using a confocal laser scanning microscope LEXT OLS4000 from Olympus (Tokyo, Japan), as shown in Figure 10. The strain gauge has a good appearance and a clear edge. The width of the strain gauge’s longitudinal branches was also measured, which was 248.106 ± 3.437 µm. The measured width is larger than the width in the design diagram. This was caused by the expansion of the PEDOT/PSS ink marks on the sensor substrate, which will deteriorate the dimensional accuracy.

**Figure 11:**
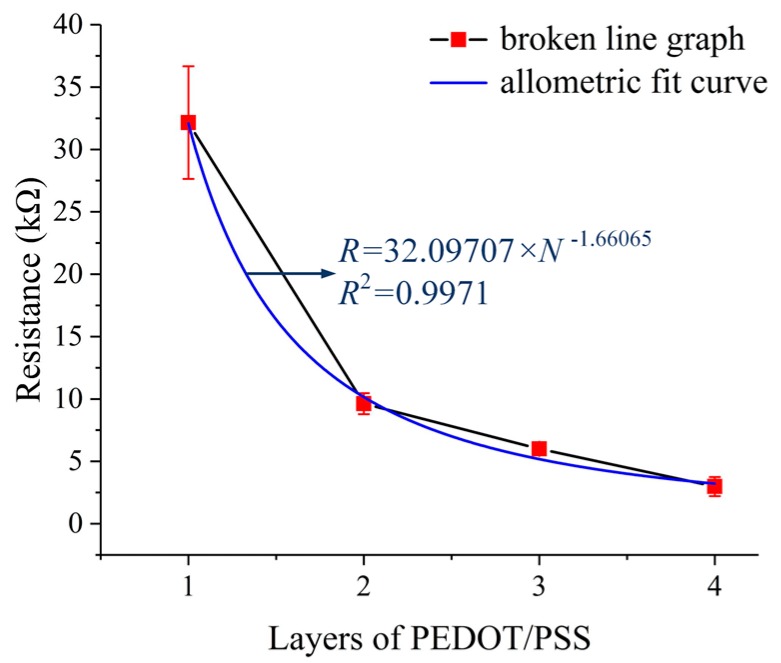
The resistance of the strain gauge versus printed layers.

The resistance of the strain gauge is an essential parameter of the strain gauge type sensor, so the strain gauge printed on the sensor substrate was also characterized. The relationship between the resistance of the strain gauge and the count of layers of PEDOT/PSS was investigated. Measurements were done using samples with 1, 2, 3, and 4 layers of PEDOT/PSS, respectively. The resistance values were measured using a FLUKE 8846A 6-1/2 digital precision multimeter (Fluke Corporation, Everett, WA, USA) at 21.7 °C, 56.0% RH. There were 20 samples (5 samples for each group) and each sample was measured three times. The experiment results are listed in Table 3. As shown in Figure 11, the resistance of the strain gauges is decreased from 32.15253 ± 3.49692 kΩ to 2.98283 ± 0.59104 kΩ as the count of layers of PEDOT/PSS was increased from 1 to 4. There is an allometric relationship between the resistance and the count of the printed layers, which is not a strict inverse relationship. Also, with the increase of the layers, the standard deviation of the resistance decreased. Therefore, the count of layers should be large enough to achieve a strain gauge with small, stable resistance. However, there was a short circuit phenomenon occurring on the strain gauge with 4 layers of PEDOT/PSS because of the expansion of the PEDOT/PSS ink marks on the sensor substrate, which deteriorates the resistance consistency. Taking all these things into consideration, the strain gauge was printed with 3 layers of PEDOT/PSS to achieve a small, stable resistance.

**Figure 12:**
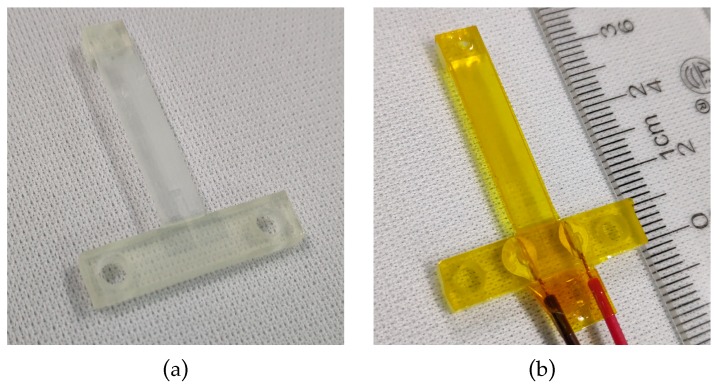
Pictures of the force sensor: (**a**) After inkjet printing; (**b**) after bonding wires and sealing.

After that, wires were connected with electrodes using a conductive silver epoxy paste from Ausbond (Shenzhen, Chian) and dried for 20 min at 120 °C. The force sensor was sealed under a layer of Kapton tape with 60 µm thickness and 5 mm width to mechanically protect the printed strain gauge and the electrical contacts. This process was done manually. The sensor substrate with the printed strain gauge is shown in Figure 12a, and the fabricated force sensor with wires and Kapton tape is shown in Figure 12b.

## 4. Test of the Force Sensor

**Figure 13:**
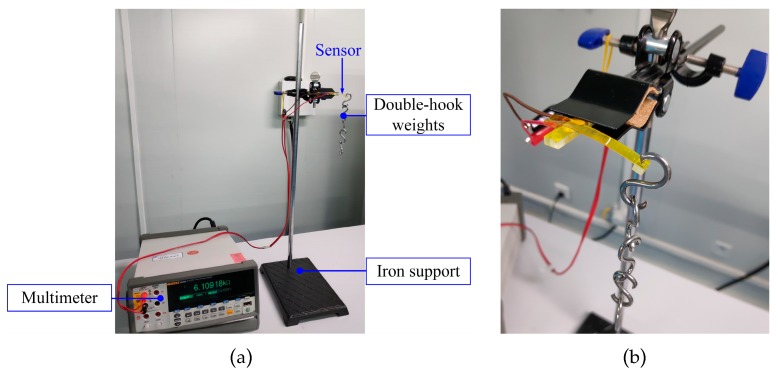
Pictures of the experimental setup: (**a**) Composition of the experimental setup; (**b**) details of the experimental setup.

At first, an experimental setup was established to characterize the force sensor. As shown in Figure 13, the experimental setup can be divided into three parts. The iron support stand was used to clamp and hold up the force sensor. The double-hook weights were utilized to apply well-defined force on the loading hole of the force sensor, and every double-hook weight is 2 g in weight with a manufactured precision of ±1.2 mg. The multimeter (FLUKE 8846A 6-1/2, Fluke Corporation, Everett, WA, USA) was used to read out the resistance of the force sensor. The experimental setup was put on the anti-vibration platform and the force sensor was clamped horizontally in an iron clip. In the static test, the loading force was applied from 0 mN increasing to 160 mN then decreasing to 0 mN with the use of eight 2 g double-hook weights. The loading and unloading process was repeated three times and mean values of the experimental data were obtained.

**Figure 14:**
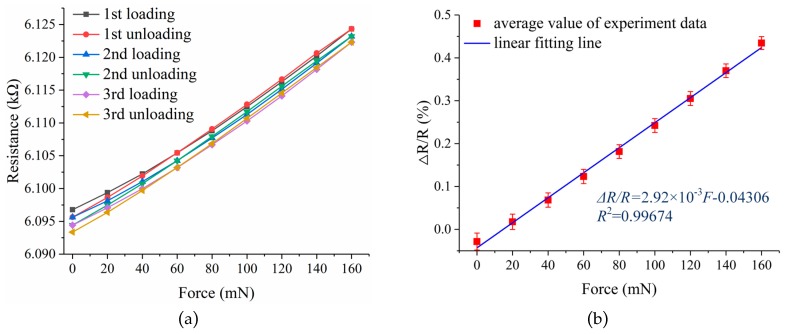
(**a**) The resistance change versus applied force; (**b**) the least square fit of experimental data.

The data of the static test are recorded in Table 4, and the results of the static test are shown in Figure 14. To better understand of the force sensor performance, the performance parameters such as sensitivity, linearity, hysteresis, repeatability, and overall accuracy were calculated. Within the range 0–160 mN, a sensitivity of 2.92% N^−1^, a linearity error of 3.1485% FS, a hysteresis of 2.0191% FS, repeatability of 6.7243% FS, and an overall accuracy of 7.6945% FS were obtained. The linearity error and repeatability error of the force sensor is relatively large, and these were mainly induced by the intrinsic properties of the high-temperature resin, such as the creep property. Combined with the simulation analysis with theoretical calculation, the effective gauge factor of the strain gauge was obtained, which was about 0.98.

**Figure 15:**
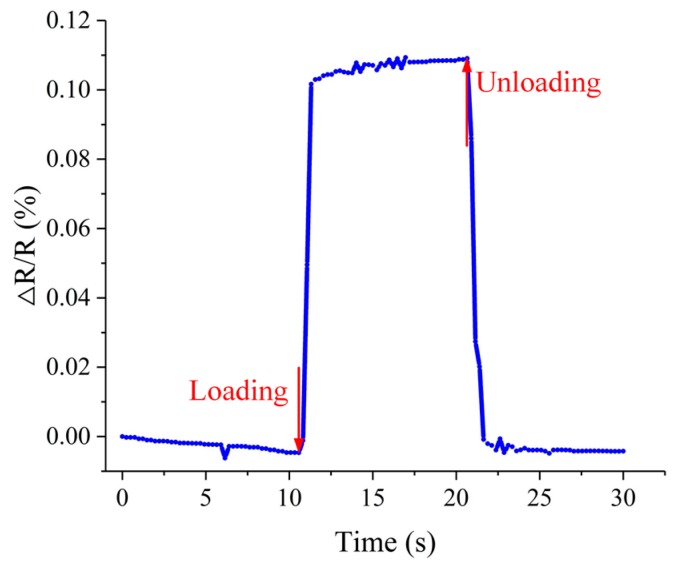
The dynamic response of the force sensor to a force pulse.

Besides the static test, the dynamic characteristics of the force sensor were also tested with the same experimental setup. The response time to a sudden increase and decrease of a force on the force sensor and the ability of the force sensor to keep a stable output under a constant force were investigated. To achieve these measurements, a 5 g double-hook weight was applied to the force sensor for a while. The whole test time was 30 s, and the loading time was about 10 s. The relative resistance variation of the force sensor under the force pulse is shown in Figure 15. The rise time was 756.1 ms the fall time was 1031.1 ms. The fall time was relatively long due to the creep property of the high-temperature resin. The fluctuation during the loading time was mainly caused by the waggle of the double-hook weight. Also, there is zero drift occurring in this test.

**Figure 16:**
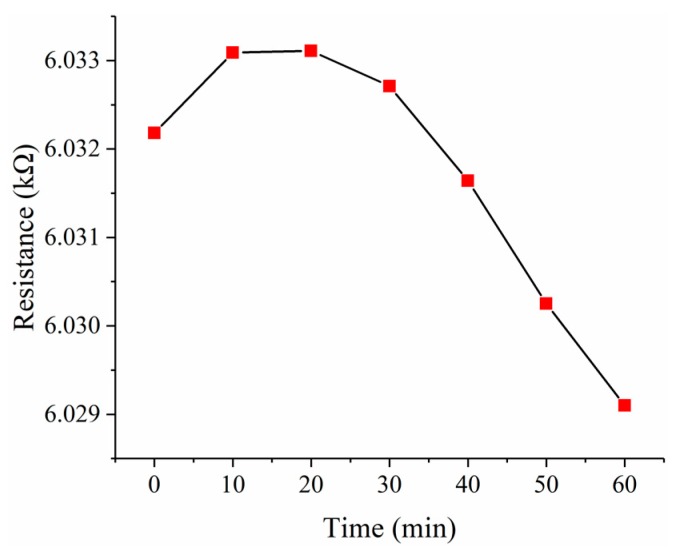
The fluctuation of the resistance in one hour.

To investigate the zero drift of the force sensor, a short-term stability test was performed for one hour. The data of the short-term stability test are recorded in Table 5. As shown in Figure 16, the resistance was read every 10 min, and the resistance changes are no more than 0.004 kΩ in one hour, which is 0.0665% of the initial resistance. The minor changes of the temperature and humidity in the laboratory may contribute to the 0.004 kΩ fluctuation.

**Figure 17:**
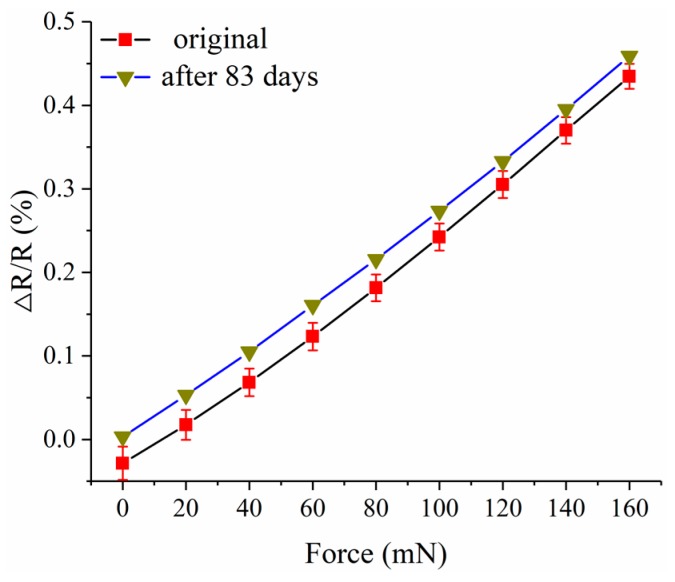
The result of the static test conducted after 83 days.

The resistance of the force sensor was measured again 80 days after the short-term stability test, and the result is 6.04316 kΩ. This may be caused by the humidity change in the laboratory, which is 48.1% RH before and 15.6% RH after. Also, the static test was conducted again after 83 days. As shown in Figure 17, the result is close to the result of the static test before, and this indicates that the long-term stability of the force sensor is good.

A cost effective and time-saving way to fabricate a force sensor with the composite structure is achieved in this research, and the force sensor has potential applications for use in robot whisker and flow velocity measurement technologies. However, the performance of the force sensor is still not good enough. The repeatability error is relatively large and the response time is long. This is because the sensor performance is somewhat restricted by the creep property of the high-temperature resin, and the resistance of the strain gauge is susceptible to temperature and humidity. Once these problems are solved, the performance can be further improved. It is this necessary to investigate new 3D printing materials and new sensor packaging methods in future studies.

## 5. Conclusions

In this paper, a novel 3D printed force sensor with an inkjet-printed strain gauge is investigated. With the combination of DLP-based printing and inkjet printing technologies, a totally 3D printed force sensor with a composite structure was proposed which has cost effective and time-saving advantages compared to traditional sensor manufacturing processes. Theoretical and simulation analyses were conducted for sensor design. To optimize process parameters, the relationship between the mechanical properties of the post-cured resin and the curing time was investigated, and the resistance of the printed strain gauges was characterized. Finally, the fabricated force sensor was tested. Experimental results indicate that the sensitivity is 2.92% N^−1^, the linearity error is 3.1485% FS, the hysteresis is 2.0191% FS, the repeatability is 6.7243% FS, and overall accuracy is 7.6945% FS in the range of 0–160 mN. The effective gauge factor of the strain gauge is about 0.98. The resistance drifting is less than 0.004 kΩ (0.0665% of the initial resistance) within an hour. These results proved that 3D printing technology may have great application potential for sensor design and fabrication. Future work will be mainly devoted to investigating new 3D printing materials and new sensor packaging methods to improve the performance of the 3D printed force sensor.

## Figures and Tables

**Table 1:** The average values and standard deviations of the experimental data.

Curing Time (Hours)	Elastic Modulus (MPa)		Flexural Strength (MPa)	
	Average	Standard Deviation	Average	Standard Deviation
0	1391.8266	156.8653	24.4600	1.0343
0.5	2834.0233	310.0489	52.3333	4.6653
1.0	2375.1133	132.4333	69.6266	9.3182
1.5	2290.9333	192.9503	79.1666	6.4207
2.0	2388.1200	130.3423	96.9066	5.8009
2.5	2105.1566	68.4182	79.6966	25.1787
3.0	2398.5733	121.3335	91.5300	4.2034

**Table 2:** Waveforms used during printing.

Parameters	Jetting Waveform				Non-Jetting Waveform		
Phase	1	2	3	4	1	2	3
Level (%)	0	100	67	40	40	40	40
Increase	0.65	0.93	0.60	0.80	1	1	1
Duration (µs)	3.584	3.712	3.392	0.832	3.712	6.975	0.832

**Table 3:** The average values and standard deviations of the resistance.

Layers of PEDOT/PSS	Resistance of the Strain Gauge (kΩ)	
	Average	Standard Deviation
1	32.15253	4.51450
2	9.62587	0.84120
3	6.00741	0.37120
4	2.98283	0.76303

**Table 4:** The data of the static test.

Force (mN)	Resistance (kΩ)					
	1st		2nd	3rd		
	Loading	Unloading	Loading	Unloading	Loading	Unloading
0	6.09678	6.09563	6.09563	6.09443	6.09443	6.09336
20	6.09938	6.09865	6.09813	6.09747	6.09707	6.09638
40	6.10224	6.10192	6.10106	6.10075	6.09999	6.09971
60	6.10544	6.10545	6.10425	6.10424	6.10319	6.10320
80	6.10880	6.10908	6.10771	6.10793	6.10668	6.10686
100	6.11247	6.11283	6.11130	6.11171	6.11030	6.11070
120	6.11627	6.11666	6.11513	6.11560	6.11411	6.11456
140	6.12020	6.12064	6.11912	6.11947	6.11816	6.11846
160	6.12433	6.12433	6.12320	6.12320	6.12230	6.12230

**Table 5:** The data of the short-term stability test.

**Time (min)**	0	10	20	30	40	50	60
**Resistance (kΩ)**	6.03218	6.03309	6.03311	6.03271	6.03164	6.03025	6.02910
